# Evaluation of Formulation Parameters on Permeation of Ibuprofen from Topical Formulations Using Strat-M^®^ Membrane

**DOI:** 10.3390/pharmaceutics12020151

**Published:** 2020-02-13

**Authors:** Pradeep Kumar Bolla, Bradley A. Clark, Abhishek Juluri, Hanumanth Srikanth Cheruvu, Jwala Renukuntla

**Affiliations:** 1 Department of Biomedical Engineering, College of Engineering, The University of Texas at El Paso, 500 W. University Ave, El Paso, TX 79968, USA; pbolla@miners.utep.edu; 2 Department of Basic Pharmaceutical Sciences, Fred Wilson School of Pharmacy, High Point University, High Point, NC 27268, USA; bclark@highpoint.edu; 3 Department of Pharmaceutics, The University of Mississippi, Oxford, MS 38677, USA; abhishek3737@gmail.com; 4 Department of Pharmaceutics, National Institute of Pharmaceutical Education and Research, Hyderabad 500037, India; cheruvusrikanth@gmail.com

**Keywords:** topical cream, topical gel, emulgel, ibuprofen, semi-solid topical formulations, permeation, Strat-M^®^, Permegear flow-through cells, formulation parameters, topical bioavailability, Quality Target Product Profile (QTPP), in-vitro permeation test (IVPT)

## Abstract

Topical drug delivery is an attractive alternative to conventional methods because of advantages such as non-invasive delivery, by-pass of first pass metabolism, and improved patient compliance. However, several factors such as skin, physicochemical properties of the drug, and vehicle characteristics influence the permeation. Within a formulation, critical factors such as concentration of drug, physical state of drug in the formulation, and organoleptic properties affect the flux across the skin. The aim of the study was to develop and investigate topical semisolid preparations (creams and gels) with ibuprofen as the model drug and investigate the effect of various formulation parameters on the in-vitro performance across the Strat-M^®^ membrane using flow-through cells. In addition, the physical stability of the developed formulations was investigated by studying viscosity, pH, and appearance. All the formulations developed in the study had appealing appearance with smooth texture and no signs of separation. Viscosity and pH of the formulations were acceptable. Cumulative amount of drug permeated at the end of 24 h was highest for clear gel (3% *w*/*w* ibuprofen; F6: 739.6 ± 36.1 µg/cm^2^) followed by cream with high concentration of ibuprofen in suspended form (5% *w*/*w*; F3: 320.8 ± 17.53 µg/cm^2^), emulgel (3% *w*/*w* ibuprofen; F5: 178.5 ± 34.5 µg/cm^2^), and cream with solubilized ibuprofen (3% *w*/*w*; F2A: 163.2 ± 9.36 µg/cm^2^). Results from this study showed that permeation of ibuprofen was significantly influenced by formulation parameters such as concentration of ibuprofen (3% vs. 5% *w*/*w*), physical state of ibuprofen (solubilized vs. suspended), formulation type (cream vs. gel), mucoadhesive agents, and viscosity (high vs. low). Thus, findings from this study indicate that pharmaceutical formulation scientists should explore these critical factors during the early development of any new topical drug product in order to meet pre-determined quality target product profile.

## 1. Introduction

Topical/transdermal drug delivery refers to the delivery of drugs via skin and is an attractive alternative to conventional methods such as oral and parenteral routes. Advantages associated with topical/transdermal delivery include non-invasive delivery, bypass of first pass metabolism, prolonged duration of action, reduced dosing frequency, constant levels of drug in the plasma, reduced drug toxicity/adverse events, improved patient compliance, and others [1,2,3]. However, skin acts as a major barrier for the entry of drugs and foreign compounds due to the presence of stratum corneum, a thin keratin-rich layer (15 µm) of dead cells embedded in an intricate lipid environment made of cholesterol, ceramides, and free fatty acids [4,5,6]. In addition, several other factors such as physicochemical properties of the drug (lipophilicity, solubility, molecular weight or size, and hydrogen bonding) and characteristics of a formulation/vehicle or a drug delivery system influence the permeation [7]. To overcome these challenges, several physical and chemical methods have been employed to enhance the transport of drugs through the skin. Physical methods include approaches such as microneedles, thermal ablation, radiofrequency, iontophoresis, ballistic liquid jets, laser, and others [4,8,9,10,11]. However, these methods are known to cause irritation to the skin due to mechanical, thermal, magnetic, and electrical energy [8]. Chemical methods include the use of penetration enhancers such as propylene glycol, ethanol, transcutol, and others to enhance the drug transport through the skin. They increase the diffusion of drugs through the skin by interacting and altering the complex structure of skin and thus enhancing the partition of drug into different layers [12,13]. Several penetration enhancers have been approved in the market, but their application in topical and transdermal formulations is limited as there is no clear understanding on how these agents enhance drug transport [14]. In addition to penetration enhancers, several other excipients/additives such as solvents, co-solvents, surfactants, humectants, thickening agents, and others are used in the development of topical/transdermal formulation. These agents act as inactive ingredients and control the extent of absorption (thermodynamic activity and partition coefficient), maintain the viscosity and pH, improve the stability as well as organoleptic properties, and increase the bulk of the formulation [15,16]. Similar to every other dosage form, topical formulation development program also involves pre-formulation development, formulation development, performance (in vitro and in vivo), and stability. A well-designed Quality Target Product Profile (QTPP) provides a structure to ensure that a formulation scientist embarks on a product development program that is efficient and yet defines a listing of all relevant medical, technical, and scientific information required to reach the desired commercial development outcome [17]. However, formulation scientists face several challenges while developing a drug product with desirable QTPP. In case of topical product development, achieving the target flux is a challenge as it is dependent of several factors.

Percutaneous drug absorption is a process which involves steps such as (i) dissolution and release of drug from the vehicle/formulation, (ii) partition of drug into the stratum corneum, (iii) diffusion of the solubilized drug across the stratum corneum, and (iv) penetration of drug into the layers of the skin [18]. The goal in the development of any topical/transdermal drug formulation is to achieve maximum flux across the skin without any drug build-up. Critical factors which influence the flux across the skin include concentration of drug in the vehicle/formulation, physical state of drug in the formulation, and other formulation properties. Concentration of drug in the formulation is important as a proportional increase in the flux can be achieved by increasing the concentration of the dissolved drug. According to Fick’s law of diffusion (Equation (1)), at a higher concentration above the solubility, the excess drug in the formulation acts as a reservoir and helps in maintaining constant flux for a prolonged period and thus increases the permeation [19]. Physical state of the drug in the formulation (solubilized drug vs. dispersed/suspended drug) can also significantly affect the permeation. It is known that greater flux is achieved when the drug is in solubilized form compared to suspended form. Enhanced permeation is attributed to increase in thermodynamic activity and partition with solubilized drug. Thus, the solubilized systems have advantages such as increased efficacy at lower concentrations, low drug irritation potential and cost efficient [16]. In addition to the above, formulation properties such as type of formulation (monophasic vs. multiphasic systems), viscosity, pH, and other organoleptic properties significantly affect the transport of drug across the skin. Therefore, pharmaceutical formulation scientists must consider all the above factors in the development of any new topical drug product. Since the inception of topical dosage forms, numerous excipients have been investigated for their application in conventional dosage forms, such as creams, gels, and ointments. Although several excipients have been approved by the United States Food and Drug Administration (USFDA) for topical use, formulation scientists face challenges in the development of topical drug product with desired permeation profile. In addition, efforts have also been made in development of novel formulations, such as microemulsions, nanoparticulate drug delivery systems, eutectic mixtures, patches, and others to enhance the permeation of drugs across the skin [20,21]. In general, there is a lack of scientific evaluation on how the formulation properties, such as concentration of drug, physical state of the drug, and formulation type, influence the bioavailability of conventional topical dosage forms, such as creams and gels. Although several studies have evaluated the effect of formulation properties on transdermal permeation of drugs, the scientific data show that the research was limited to only one factor, such as concentration of drug [22], concentration of excipients [7,23], and formulation type [24,25]. Therefore, the current study was designed to provide ready information to the formulators who design and develop topical semisolid formulations on the impact of formulation properties (concentration of drug, physical state of the drug, mucoadhesive agents, and formulation type) on transdermal permeation.

(1) $$J=K\cdot \Delta C=\frac{D\cdot K_{p}\cdot \Delta C}{h}$$

Equation (1): Fick’s law of diffusion, where *J* is the steady-state flux of the drug molecule through the skin (µg/cm^2^·h), *K* is the permeability coefficient (cm/s), Δ*C* is the difference in concentration (µg/cm^3^), *D* is the diffusion coefficient (cm^2^/s), *K_p_* is the apparent partition coefficient, and *h* is the thickness of the layer of skin (cm).

Non-steroidal anti-inflammatory drugs (NSAIDs) are used to treat local pain and inflammation associated with injuries, rheumatoid arthritis, osteoarthritis, and other musculoskeletal problems [7,26]. Although, NSAIDs are very effective, their oral absorption is associated with severe gastric irritation leading to gastric bleeding and ulcers. Therefore, topical/transdermal delivery of NSAIDs is preferred as it bypasses hepatic first pass metabolism and also results in targeted effect at the site of inflammation/pain [24]. Majority of the NSAIDs (salicylates, acetic acid derivatives, enol acid derivatives, and propionic acid derivatives) approved by USFDA have similar physicochemical properties (molecular mass, log*P,* and p*K*_a_) [27,28,29,30]. Hence, it can be assumed that there may be similarities in transdermal permeation for these compounds [29]. Among these agents, ibuprofen is the most commonly used NSAID. Ibuprofen (α-methyl-4-(2-methylpropyl) benzeneacetic acid) is a weak acid (p*K*_a_ 4.5–4.6), thus the pH of the skin (~4.8) favors passive diffusion as majority of the molecules will be in unionized form. However, poor aqueous solubility (0.084 and 0.685 mg/L at pH 4.5 and 5.54, respectively) limits the skin permeation of ibuprofen. Ibuprofen is considered as an attractive candidate for topical/percutaneous delivery due to the physicochemical properties (low molecular weight (MW: 206.29 g·mol^−1^), suitable partition coefficient (log*P*: 3.68), and short elimination half-life (*t*_1/2_ 2–4 h), [7]. Currently, topical formulations of ibuprofen are not approved in the United States. Therefore, taking into account all the above factors and availability of drug for research purposes, ibuprofen was chosen as the model drug for our study. The main goal of the study was to prepare semisolid formulations and investigate the effect of concentration of drug, formulation type and physical state of drug on transdermal permeation of ibuprofen. All the excipients (except Sepineo SE 68) used were approved by the USFDA for topical use and were within the limits listed in the inactive ingredient database. In the present study, we have developed ibuprofen topical creams at two concentrations (3% and 5% *w*/*w*)—emulgel (3% *w*/*w*) and clear non-aqueous gel (3% *w*/*w*). Further, in-vitro permeation studies were performed across the Strat-M^®^ membrane to study the effect of various formulation parameters on the permeation of ibuprofen.

## 2. Materials

Ibuprofen, hydroxy propyl methyl cellulose (HPMC) (MW: 86,000, viscosity 4000 cps at 2% solution), and hydroxy propyl cellulose were purchased from Acros Organics (Fair Lawn, NJ, USA). Absolute ethanol, sorbitan monolaurate (Span 20), sodium chloride, glacial acetic acid, acetonitrile (HPLC grade) were procured from Fisher Chemicals (Fair Lawn, NJ, USA). Deionized water used in all the experiments was obtained from in-house Milli-Q^®^ IQ 7000 Ultrapure Water System (EMD Millipore, Bedford, MA, USA). Mineral oil NF and white petrolatum were purchased from PCCA (Houston, TX, USA). Tefose^®^ 63 (mixture of PEG-6 stearate NF/JPE and Ethylene glycol palmitostearate EP/NF/JPE) and Transcutol^®^ (Diethylene glycol monoethyl ether EP/NF) were gift samples from Gattefossé (Paramus, NJ, USA). Kollicream^®^ IPM (isopropyl myristate), Kollicream^®^ OA (oleyl alcohol), Kollisolv^®^ MCT 70 (medium-chain triglycerides), Kollisolv^®^ PEG 400 (polyethylene glycol 400), Kollisolv^®^ PG (propylene glycol), Kolliphor^®^ CS 20 (macrogol cetostearyl ether 20/polyoxyl 20 cetostearyl ether), Kolliphor^®^ PS 80 (polysorbate 80), Kolliphor^®^ CS A (cetostearyl alcohol (type A)), Kolliwax^®^ CA (cetyl alcohol), and Kolliwax^®^ SA (stearyl alcohol) were generous samples from BASF (Tarrytown, NY, USA). Glycerol monostearate was obtained from Alfa Aesar (Ward Hill, MA, USA). Strat-M^®^ membrane and glycerol were procured from Sigma-Aldrich (St. Louis, MO, USA). Carbopol 974P (Carbomer Homopolymer Type B) was a sample from Lubrizol Life Sciences (Cleveland, OH, USA). Sepineo™ P600 (acrylamide/sodium acryloyldimethyl taurate copolymer/isohexadecane and Polysorbate 80) and Sepineo™ SE 68 (cetearyl alcohol, cetearyl glucoside) were gift samples from Seppic Inc (Fairfield, NJ, USA).

## 3. Methods

### 3.1. Solubility of Ibuprofen in Solvents

The solubility of ibuprofen in liquid excipients was determined using visual solubility protocol. In this method, the excipients were accurately weighed (2.5 g) in individually-labelled 20 mL scintillation vials. To these vials, accurately weighed aliquots of ibuprofen (~5 mg for glycerol due to poor solubility and ~25 mg for other excipients) was added and tightly closed. Further, the vials were placed in a shaking water bath (Fisher Scientific, Waltham, MA, USA) maintained at 25 °C for at least 15 min to allow proper mixing. After 15 min, the vials were visually inspected, and additional aliquots of ibuprofen were added periodically (every 15 min) until saturation was achieved. Following this, the vials were placed in the shaking water bath for 24 h and visually inspected the following day. The final weight of the vials was measured to determine the approximate solubility of ibuprofen in each excipient and reported as mg/g and percentage (%).

### 3.2. Formulation of Ibuprofen Creams and Gels

#### 3.2.1. Optimization of Formulations

Optimization of all the formulations was performed by evaluating the effect of different concentrations of excipients on the stability, excipient instability, viscosity, and any visual changes in the formulations (Appendix A). After optimization, stable formulations which provided acceptable appearance and viscosity were chosen for further evaluation. Compositions of all the optimized creams, emulgel and clear gel are provided in Table 1, Table 2, and Table 3, respectively. All the formulations had differences in composition since the main aim of this study was to evaluate on how formulation parameters such as concentration and physical state of the drug, formulation type and mucoadhesive agents influence the permeation of ibuprofen.

#### 3.2.2. Formulation of Creams

Compositions of the creams developed is provided in Table 1. Ibuprofen creams were developed at two different strengths (3% *w*/*w* (F1A, F1B, F2A, F2B) and 5% *w*/*w* (F3, F4)) using a water-in-oil (w/o) emulsion method. Briefly, ibuprofen was accurately weighed and transferred into a 250 mL beaker containing all the required oil phase components for each formulation. In another 100 mL beaker, accurately weighed water soluble components were dissolved in water. Both the beakers were placed in a water bath and heated to 65 ± 2 °C. Once both the phases reached approximately similar temperature, the aqueous phase was added to the oil phase and homogenized at 5000 rpm using a high shear homogenizer (Fisherbrand™ 850, Waltham, MA, USA) for 10 min to form an emulsion. After homogenization, the emulsion was allowed to cool down to room temperature by mixing it using an overhead stirrer (IKA RW20 Digital, Wilmington, NC, USA) at 300 rpm for 2 h until a smooth cream was obtained. Sepineo P600 was added to the mixture during the process of homogenization for formulations F3 and F5.

#### 3.2.3. Formulation of Emulgel

Composition of ibuprofen emulgel (F5) (3% *w*/*w*) is provided in Table 2. Ibuprofen (3 g) was dissolved in ethanol (30 mL) and water was added to ibuprofen solution. To this mixture, accurately weighed Sepineo P600 was added immediately and vigorously mixed using a glass rod until a smooth emulgel was formed.

#### 3.2.4. Formulation of Clear Non-Aqueous Gel

Composition of ibuprofen clear non aqueous gel (F6) (3% *w*/*w*) is provided in Table 3. Ibuprofen was weighed and added to a mixture of propylene glycol, ethanol, transcutol, and glycerin to obtain a clear solution. To this clear solution, PEG 400 was added and mixed on a magnetic stirrer. Accurately weighed HPC (4 g) was dispersed in the mixture and allowed to thicken at room temperature using an overhead mixer (IKA RW20 Digital, Wilmington, NC, USA) at 500 rpm for 2 h.

### 3.3. Polarized Light Microscopy

Polarized light microscopy was used to study the microscopic features of the optimized creams and gels. All the formulations were applied on a microscopic glass slide and evenly spread with a coverslip. The cover slipped slides were observed under Amscope^®^ PZ300 series polarized light microscope (Amscope, Irvine, CA, USA) in the transmission mode at 180× magnification and photomicrographs were captured on a laboratory PC.

### 3.4. HPLC Analysis of Ibuprofen

The amount of ibuprofen in the samples was quantified using Waters Alliance e2695 HPLC equipped with 2998 photodiode array detector and Empower 3.0 software. The analysis was carried out on a reverse phase Phenomenex^®^ C_18_ column (250 × 4.6 mm; 5 µm particle size) at 25 °C. The mobile phase was a mixture (60:40) of acetonitrile and water (adjusted to pH 3.8 with acetic acid) at a constant flow rate of 1.5 mL/min. Samples (60 µL) were injected into the column using autosampler and monitored at 220 nm. Retention time of ibuprofen was 6.5 min. All the samples injected were filtered through 0.45 µm membrane filter.

### 3.5. Measurement of Viscosity and pH

Rheological experiments were conducted to measure the viscosity of the optimized formulations. Measurements were performed at room temperature using a Viscolead-one digital viscometer (Fungilab Inc. New York, NY, USA) equipped with a spindle rotor (R6) set at 20 rpm. The method was validated by using 2% HPMC gel (4000 cps) as a control. The pH of the formulations was evaluated on day 0 and day 60 using a calibrated Mettler Toledo InLab^®^ pH meter equipped with LE422 micro pH electrode (Mettler Toledo, Columbus, OH, USA).

### 3.6. In-Vitro Permeation Studies

**Figure 1:**
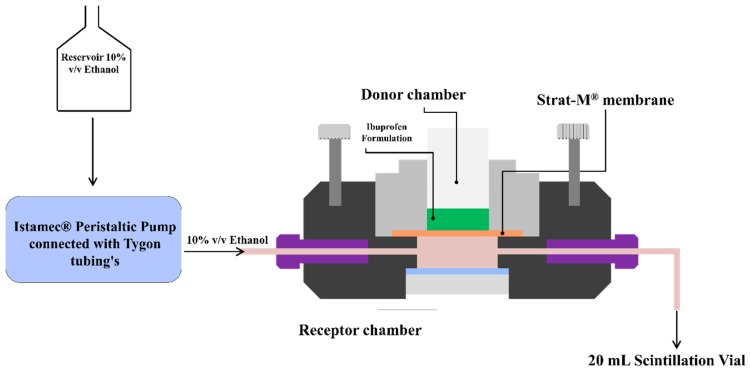
Permegear ILC-07^®^ automated flow-through cells.

In-vitro permeation studies were conducted using a PermeGear® ILC-07 automated system (PermeGear, Riegelsville, PA, USA) equipped with seven in-line flow-through diffusion cells, made of Kel-F. Each diffusion cell had a donor and receptor chamber clamped with threaded rods and adjustable locking nuts. Receptor chambers (volume: 254 µL receptor) had inlet and outlet ports connected to the Tygon tubings having 1/4-28 HPLC fittings. Temperature of the cells was maintained at 32 °C using Julabo BC4 circulating water bath (Seelbach, Germany). Diameter of the diffusional area was 1 cm (total diffusional area: 0.785 cm^2^) and the cells were connected to a 7-channel peristaltic pump® IPC (Ismatec, Zurich, Switzerland) which draws receptor solution from a reservoir (Figure 1). Strat-M^®^ was used as a diffusion membrane, which was mounted on the cells and sandwiched between the donor and receptor chambers using the adjustable locking nuts. Formulations (~10 mg ibuprofen) were placed on the diffusion membrane and the receptor fluid (10% *v*/*v* ethanol) was allowed to flow at a rate of 4 mL/h for 24 h. At pre-determined time intervals, the receptor fluid was collected in 20 mL scintillation vials and analyzed using HPLC to determine the amount of ibuprofen permeated through the Strat-M^®^ membrane.

### 3.7. Permeation Data Analysis

The permeation profile from the formulations was plotted as cumulative amount of ibuprofen permeated vs. time. The flux (µg/cm^2^/h) and lag-time (h) estimates were generated using Skin and Membrane Permeation Data Analysis (SAMPA) software, version 1.04, a free software tool used for skin and membrane permeation data analysis [31].

### 3.8. Physical Stability

Physical stability studies were conducted for all the formulations at 25 ± 2 °C and at 40 ± 2 °C. All the samples were transferred to glass scintillation vials, closed tightly and stored at 25 ± 2 °C and 40 ± 2 °C. Samples were evaluated for stability, changes in color, and any other physical instability for 90 days.

### 3.9. Statistical Analysis

All the data was statistically analyzed using GraphPad Prism software (Version 5.0, San Diego, CA, USA). Permeation data analysis was performed using SAMPA software, version 1.04. A *p*-value of <0.05 was considered as statistically significant.

## 4. Results and Discussion

### 4.1. Solubility in Solvents

The solubility of ibuprofen in different solvents is provided in Table 4. Results show that transcutol and propylene glycol provided the greater solubility (300 mg/g), whereas glycerol provided lowest solubility of ibuprofen (4 mg/g). The order of ibuprofen solubility in various solvents was transcutol = propylene glycol > isopropyl myristate > polyethylene glycol 400 > oleyl alcohol = polysorbate 80 > medium chain triglycerides > mineral oil > glycerol. Results from the solubility studies are in agreement with the literature where solvents/co-solvents such as transcutol, propylene glycol, oleyl alcohol, and isopropyl myristate enhance the solubility of poorly-soluble compounds [1].

### 4.2. pH and Viscosity

All the optimized formulations developed in the study had appealing appearance with smooth texture and no signs of phase separation. Physical evaluation was done by pressing a small quantity of formulation between the thumb and index finder. It was observed that all the formulations were homogeneous and consistent without any coarse particles. The color of all the creams and emulgel was observed to be white to translucent white (Table 5). Viscosity is an important factor for semisolid formulations as it may influence the release of drug by altering the diffusion rate from the vehicles. Results for the viscosity and pH of all the formulations are provided in Table 5. Viscosity of the formulations ranged from 1872 to 32,655 cps. There was no significant change in the pH of the formulations over 60 days. The range of pH of the formulations was 4.2 to 5.95, which is close to the pH of human skin, hence there is minimal risk of skin irritation expected. In addition all excipients used in the formulations were approved by the USFDA (except Sepineo SE 68) for dermatological applications, and the concentrations used were within the limits listed in the inactive ingredients database for approved drug products [32]. Thus, there is minimal potential risk expected of skin drying, sensory reactions, and alterations in skin hydration with the formulations.

### 4.3. Polarized Light Microscopy

**Figure 2:**
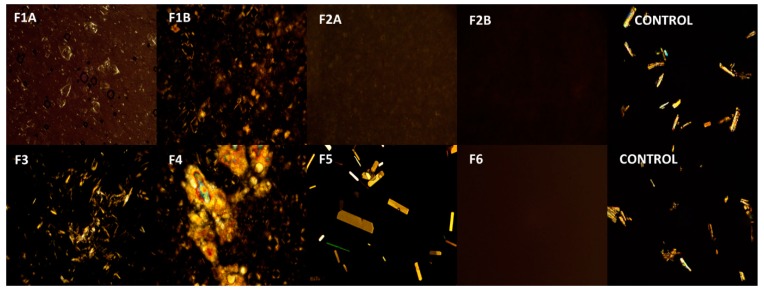
Polarized light microphotographs of formulations. Images were captured using Amscope polarized light microscope at 180× magnification. F1A, ibuprofen suspended cream (3% *w*/*w*) with Tefose 63, F1B, ibuprofen suspended cream (3% *w*/*w*) without Tefose 63, F2A, ibuprofen solubilized cream (3% *w*/*w*) with HPMC, F2A, ibuprofen solubilized cream (3% *w*/*w*) without HPMC, F3, ibuprofen suspended cream (5% *w*/*w*), F4 ibuprofen suspended cream (5% *w*/*w*), F5, ibuprofen emulgel (3% *w*/*w*), F6, ibuprofen clear non aqueous gel (3% *w*/*w*).

Polarized light microscopy was used to study the presence of ibuprofen particulates in the formulations. Polarized light microphotographs of all the formulations and control (ibuprofen in mineral oil) are provided in Figure 2. Table 6 summarizes the observations from polarized light microphotographs. It can be observed from the images that ibuprofen is in solubilized form (no crystals) in formulations F2A, F2B, and F6, whereas in all the other formulations presence of crystals in the images indicate that the drug was suspended in the formulation. Moreover, presence of a higher number of ibuprofen crystals in formulations F3 and F4 is due to the higher concentration of ibuprofen in F3 and F4 (5% *w*/*w*) compared to other formulations (3% *w*/*w*).

### 4.4. In-Vitro Permeation Studies

**Figure 3:**
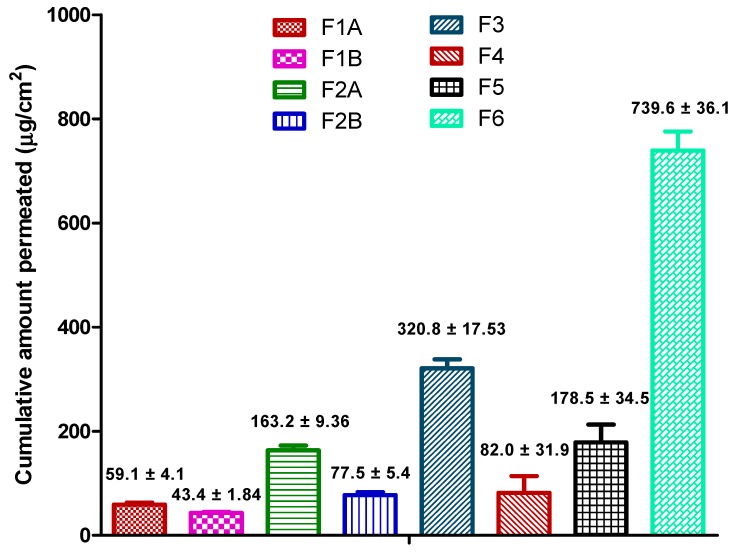
Cumulative amount of drug permeated from the formulations at the end of 24 h.

Cumulative amount and flux of drug permeated through the Strat-M^®^ membrane at the end of 24 h for all the formulations is provided in Figure 3 and Table 7, respectively. Recently, there has been a significant rise in using synthetic artificial membranes (cellulose acetate, Strat-M^®^, Parallel Artificial Membrane Permeability Assay (PAMPA)) and 3-D cultured human skin models as an alternative to human and animal skin in the development of topical and transdermal formulations [33]. In 2018, European Medicines Agency’s draft guideline on quality and equivalence of topical products has recommended the use of synthetic membranes to better understand and characterize performance of a finished topical dosage form [34]. Moreover, synthetic membranes are inexpensive and easily resourced with superior data reproducibility [35,36]. Therefore, for our studies, Strat-M^®^ was used as a diffusion membrane. Strat-M^®^ is a multilayered synthetic membrane (300 µm thickness) similar to skin and made up of several tightly-packed layers of polyester sulfone. Several studies have been reported in the literature comparing the ability of Strat-M^®^ membrane to predict the permeation of hydrophilic and lipophilic compounds such as diclofenac, hydrocortisone, caffeine, amphotericin B, and capsaicin. Results have shown that the Strat-M^®^ membrane had better correlation to human skin with minimal lot-to-lot variability, safety, and storage limitations [37,38,39]. Uchida et al. evaluated the skin permeabilities of 13 chemical compounds using Strat-M^®^ membrane and compared them to human and animal skins. Results confirmed that permeability coefficients, diffusion, and partition parameters were well correlated between the Strat-M^®^ membrane and human and animal skin [33]. Recently, Haq et al. compared the Strat-M^®^ membrane with human skin on permeation of nicotine. Results showed that there was again a high correlation between human skin and Strat-M^®^ with R^2^ value of 0.99 [40]. Hence, we used Strat-M^®^ as a diffusion membrane for our studies.

#### 4.4.1. Effect of Mucoadhesive Agents (F1A vs. F1B and F2A vs. F2B)

As per the formulation composition (Table 1), F1A and F1B, F2A and F2B had the same surfactant system and concentration of ibuprofen but the only difference was the addition of mucoadhesive agents such as Tefose 63 in F1A and HPMC in F2A. Permeation data show that the addition of mucoadhesive agents in the formulations (F1A and F2A) resulted in higher permeation of ibuprofen (F1A: 59.1 ± 4.1 µg/cm^2^ vs. F1B: 43.4 ± 1.84 µg/cm^2^ and F2A: 163.2 ± 9.36 µg/cm^2^ vs. 77.5 ± 5.4 µg/cm^2^). Addition of mucoadhesive agents in the formulation could have resulted in increased retention, prolonged contact, and reduced leakage of formulation and thus increasing the permeation. Additionally, these agents increase level of hydration in the membrane interface which in turn decreases the diffusional path length and thus favoring the transport of ibuprofen [24,41]. In addition, mucoadhesive agents could have increased the concentration gradient due to prolonged contact of the formulation at the membrane interface and resulting in enhanced permeation [42,43]. Addition of mucoadhesive agents could be advantageous in the development of topical formulations intended for vaginal applications where bio-adhesion of mucoadhesive polymers, such as tefose 63 and HPMC, increase the retention and absorption of drugs [44,45,46].

#### 4.4.2. Effect of Physical State of the Drug (F1A vs. F2A)

Physical state of drug in the formulation (solubilized vs. suspended) could significantly affect the release of drug from the formulation. Polarized light microscopy (Figure 2) revealed that even though ibuprofen concentration was the same (3% *w*/*w*) in F1A and F2A, drug was in solubilized form in F2A and suspended form in F1A. Permeation results show that cumulative amount of drug permeated at the end of 24 h was significantly higher in F2A compared to F1A (163.2 ± 9.36 µg/cm^2^ vs. 59.1 ± 4.1 µg/cm^2^). Enhanced permeation in F2A could be attributed to increased thermodynamic activity with the solubilized formulation. Additionally, ibuprofen could have been dissolved in the oil phase resulting in no dispersed drug particles in the formulation. Thus, solubilized drug increased the partition into the membrane and thus resulted in enhanced permeation [16]. These types of systems are suitable for enhanced permeation of lipophilic drugs, like ibuprofen, which tend to dissolve in the oil phase and partition across the skin.

#### 4.4.3. Effect of Drug Concentration in the Formulation (F1A vs. F3 and F2A vs. F4)

##### F1A vs. F3

As per the formulation composition table provided in the earlier section (Table 1), F1A and F3 had the same surfactant system to stabilize the formulation, but F3 had higher concentration of drug (5% *w*/*w*) compared to F1A (3% *w*/*w*). Polarized light microscopy results revealed that ibuprofen existed in a suspended form in both the formulations (Figure 2). As expected, permeation data showed that cumulative amount of drug ibuprofen permeated at the end of 24 h was significantly higher with F3 compared to F1A (320.8 ± 17.53 µg/cm^2^ vs. 59.1 ± 4.1 µg/cm^2^). Enhanced permeation of ibuprofen could be attributed to the higher concentration of ibuprofen in F3, which acted as a reservoir and helped in maintaining concentration gradient for a prolonged period [19]. These types of systems should be considered for drugs that require high doses, such as NSAIDs (ibuprofen and diclofenac) and lidocaine, where the goal is to achieve maximum flux across the skin for improving efficacy.

##### F2A vs. F4

As per the formulation composition table (Table 1) provided in the earlier section, F2A and F4 had the same surfactant system to stabilize the cream formulation but F4 had higher concentration of drug (5% *w*/*w*) compared to F2A (3% *w*/*w*). Polarized light microscopy results revealed that ibuprofen existed in a suspended form in F4 and solubilized form in F2A (Figure 2). Interestingly, permeation results showed that although F4 had higher ibuprofen concentration compared to F2A (5% *w*/*w* vs. 3% *w*/*w*), cumulative amount of drug permeated at the end of 24 h was less compared to F2A (82.0 ± 31.9 µg/cm^2^ vs. 163.2 ± 9.36 µg/cm^2^). Enhanced permeation with F2A formulation could be attributed to increased thermodynamic activity and partition of the solubilized drug in the formulation [47]. Solubilized systems are preferred for highly-potent drugs such as fentanyl, progesterone, and testosterone, requiring very less concentration of drug for activity. Formulations with highly-potent drugs have a high risk if there is residual drug leftover after the intended use period, which may impact the product’s quality, efficacy, and safety. Recently USFDA has released a guidance on minimizing the residual drug in the formulations. One of the recommendations was to design formulations with optimal drug delivery and minimal residual drug [48]. Thus, the approach of using systems with solubilized drugs could minimize the residual drug and maintain the desired permeation rate of drugs throughout the usage period.

#### 4.4.4. Effect of Formulation Type

##### Solubilized Cream vs. Non-Aqueous Gel (F6 vs. F2A)

Although the concentration of ibuprofen was similar and the drug was in solubilized form in formulations F6 and F2A, cumulative amount of drug permeated was significantly higher with gel compared to solubilized cream (F6: 739.6 ± 36.1 µg/cm^2^ vs. F2A: 163.2 ± 9.36 µg/cm^2^). Enhanced permeation could be due to low viscosity of the gel which resulted in high drug release from the formulation. Moreover, higher concentration of permeation enhancers in the formulation could have increased the solubility of ibuprofen and enhanced the diffusivity across the membrane.

##### Emulgel vs. Cream (F5 vs. F1A and F2A)

Permeation data show that cumulative amount of drug permeated at 24 h was higher with emulgel even though ibuprofen was available as suspended form in the formulation (F5: 178.5 ± 34.5 µg/cm^2^ vs. F2A: 163.2 ± 9.36 µg/cm^2^ vs. F1A: 59.1 ± 4.1 µg/cm^2^). Emulgels, also known as emulsified gels, are biphasic systems, containing aqueous gel dispersed with lipid phase, and are closely related to cream. It is reported in the literature that emulgels provide better solubility for poorly water-soluble drugs like ibuprofen and enhance the skin permeability. Higher permeation of ibuprofen with emulgels compared to creams could be attributed to increased thermodynamic activity of ibuprofen, due to a change in the vehicle where ibuprofen was more soluble, or due to a different interaction of the formulation with the membrane [24,49,50].

##### Clear Gel vs. Emulgel (F6 vs. F5)

At the end of 24 h, significantly higher amount of ibuprofen was permeated across the membrane with clear non aqueous gel (739.6 ± 36.1 µg/cm^2^) compared to emulgel (178.5 ± 34.5 µg/cm^2^). Although, concentration of ibuprofen in the formulation was similar in both the formulations, low viscosity and presence of ibuprofen in solubilized form in the gel formulation resulted in enhanced permeation. As discussed earlier, low viscosity of the clear gel resulted in greater permeation due to immediate and higher release of ibuprofen from formulation. Additionally, high thermodynamic activity and partition of ibuprofen could have resulted in enhanced permeation.

### 4.5. Permeation Data Analysis

**Figure 4:**
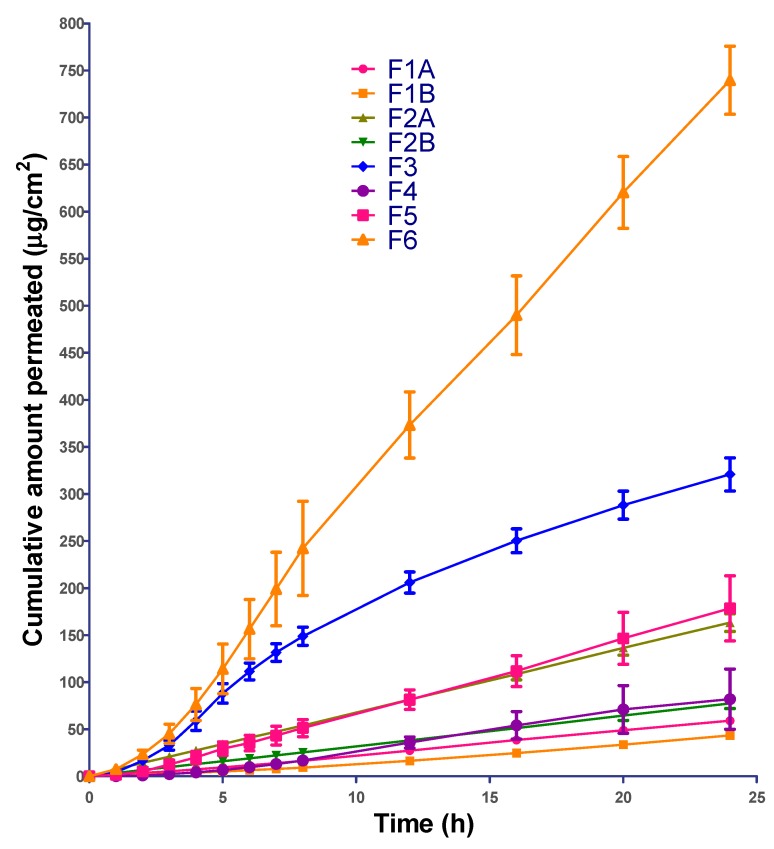
In-vitro permeation profile of all the formulations.

The cumulative amount of ibuprofen permeated was plotted as a function of time and the permeation profiles are shown in Figure 4. All the formulations showed significant lag in ibuprofen permeation except F2A and F2B. This could be due to the presence of drug in solubilized form in F2A and F2B (Table 7).

### 4.6. Physical Stability

**Figure 5:**
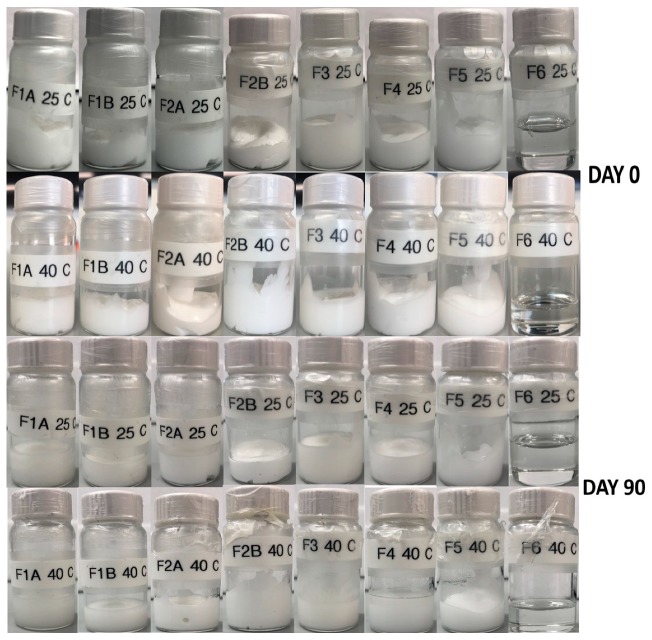
Physical stability of creams and gels at 25 and 40 °C.

All the formulations were stored at 25 ± 2 °C and 40 ± 2 °C to observe any changes in the physical stability. As shown in the Figure 5, there were no observable changes in color and stability (phase separation, creaming, crystallization, and phase inversion) of all creams and gels. This suggests that all the formulations were physically stable throughout the storage period at both temperatures.

## 5. Conclusions

Formulation of a topical drug product for lipophilic drugs with desirable QTPP is challenging. Several factors such as physicochemical properties of the drug, formulation parameters, excipients in the formulation, and other parameters can affect the permeation profile. For successful topical product development, a thorough understanding of the impact of these factors on product performance is required. This study gives an example of a screening study approach that evaluates several excipients and formulation variables on product performance. It is already known that active drug accounts only for a minor fraction in the formulation and; therefore, it is important to understand how the excipients within the formulation interact with the active drug and influence the permeation. The aim of the present study was to investigate the effect of formulation parameters, such as concentration of drug, physical state of the drug, addition of mucoadhesive agents, and formulation type, on permeation of ibuprofen from semisolid formulations. For this, we successfully developed eight different semisolid formulations (creams, emulgel and gel) of ibuprofen and evaluated the effect of formulation parameters on the in-vitro permeation. All the formulations were of acceptable quality and remained physically stable over a period of 90 days at room temperature and 40 °C. Results from the present investigation noted considerable differences in permeation of ibuprofen across the Strat-M^®^ membrane due to significant influence of formulation parameters, including concentration of ibuprofen (3% vs. 5% *w*/*w*), physical state of ibuprofen (solubilized vs. suspended), formulation type (cream vs. gel), mucoadhesive agents, and viscosity (high vs. low). It is clear according to the permeation profile that F6 (3% *w*/*w* ibuprofen clear non-aqueous gel) had the highest permeation rate among all formulations evaluated. Thus, findings from this study indicate that pharmaceutical formulation scientists should explore these critical factors during the early development of any new topical drug product in order to meet pre-determined QTPP. Additionally, as the majority of the NSAIDs have similar physicochemical properties, this article could serve as a ready reference to formulation scientists for selection of the formulation type to achieve desirable permeation profile.

## Figures and Tables

**Table 1:** Composition of optimized creams in the study.

Components	Formulation					
	F1A (%*w*/*w*)	F1B (%*w*/*w*)	F2A (%*w*/*w*)	F2B (%*w*/*w*)	F3 (%*w*/*w*)	F4 (%*w*/*w*)
Ibuprofen	3	3	3	3	5	5
Propylene glycol	15	15	-	-	-	-
Oleyl alcohol	-	-	5	5	-	-
Isopropyl myristate	-	-	-	-	10	-
Transcutol	-	-	-	-	-	15
Mineral oil	10	10	-	-	7	-
White petrolatum	-	-	10	10	-	-
Polyethylene glycol 400	-	-	-	-	10	-
Medium chain triglycerides	-	-	-	-	-	10
Glycerol	-	-	-	-	-	5
Span 20	3.8	3.8	-	-	4.7	-
Kolliphor polysorbate 80	3.3	3.3	-	-	1.3	-
Kolliphor CS 20	-	-	4.1	4.1	-	5
Glycerol monostearate	-	-	2.5	2.5	-	1.1
Cetyl alcohol	7	7	-	-	-	7
Stearyl alcohol	-	-	10	10	-	-
Cetostearyl alcohol	-	-	-	-	5	-
Sepineo SE68	5	5	-	-	-	-
Sepineo P600	-	-	-	-	4	-
Tefose 63	5	-	-	-	8	-
HPMC	-	-	1	-	-	-
Carbopol 974	-	-	-	-	-	0.5
Water	47.9	52.9	64.4	65.4	45	51.4

**Table 2:** Composition of optimized Emulgel in the study.

Components	Formulation
	F5 (%*w*/*w*)
Ibuprofen	3
Ethanol	30
Sepineo P600	4
Water	63

**Table 3:** Composition of optimized clear non-aqueous gel in the study.

Components	Formulation
	F6 (%*w*/*w*)
Ibuprofen	3
Propylene glycol	15
Ethanol	10
Glycerol	10
Transcutol	15
Hydroxy propyl cellulose	4
Polyethylene glycol 400	43

**Table 4:** Visual solubility of ibuprofen in solvents.

Excipient	Ibuprofen Solubility in Excipient (mg/g)	Percentage (%)
Transcutol	300	30
Propylene glycol (Kollicream® PG)	300	30
Isopropyl myristate	200	20
Oleyl alcohol (Kollicream® OA)	180	18
MCT 70 (medium chain triglycerides)	140	14
Mineral oil	60	6
PEG 400	190	19
Glycerol	4	0.4
Kolliphor® PS 80	180	18

**Table 5:** Viscosity and pH of ibuprofen creams and gels.

Formulation	Appearance	Viscosity (cps) (Day 0)	Initial pH (Day 0)	pH (Day 60)
F1A	Smooth white cream	23,451	4.44	4.49
F1B	Smooth white cream	23,626	5.06	5.1
F2A	Smooth white cream	32,655	4.45	4.57
F2B	Smooth white cream	18,954	4.57	4.46
F3	Smooth off-white cream	11,916	4.47	4.25
F4	Smooth white cream	11,500	4.22	4.29
F5	Smooth translucent emulgel	29,659	4.51	4.3
F6	Clear gel	1872	5.95	5.84

**Table 6:** Observations from polarized light microscopy.

Formulation	Observations
F1A	Faint white crystals of ibuprofen suspended in the cream
F1B	Faint white crystals of ibuprofen suspended in the cream
F2A	No evidence of ibuprofen crystals indicating solubilized ibuprofen in the cream
F2B	No evidence of ibuprofen crystals indicating solubilized ibuprofen in the cream
F3	High percentage of ibuprofen crystals suspended in the cream due to high concentration of drug
F4	Clear evidence of ibuprofen crystals in the cream
F5	Rod like ibuprofen crystals suspended in the emulgel
F6	No evidence of ibuprofen crystals indicating solubilized ibuprofen in the gel

**Table 7:** Cumulative amount, flux, and lag-time of drug permeated from the formulations at the end of 24 h (mean ± SD).

Formulation	Cumulative Amount Permeated at 24 h (µg/cm^2^)	Flux (µg/cm^2^/h)	Lag-Time (h)
F1A	59.1 ± 4.1	2.70 ± 0.1	1.62 ± 0.3
F1B	43.4 ± 1.84	2.25 ± 0.08	4.92 ± 0.09
F2A	163.2 ± 9.36	7.44 ± 0.41	No lag
F2B	77.5 ± 5.4	3.63 ± 0.22	No lag
F3	320.8 ± 17.53	21.19 ± 0.94	1.05 ± 0.22
F4	82.0 ± 31.9	5.39 ± 1.37	5.17 ±1.5
F5	178.5 ± 34.5	7.41 ± 0.45	1.74 ± 0.67
F6	739.6 ± 36.1	37.25 ± 5.1	2.37 ± 0.58

## Appendix A

### Optimization of Formulations

Results for the optimization of ibuprofen formulations are provided in Table A1, Table A2 and Table A3. The goal of the optimization experiments was to formulate creams and gels with acceptable appearance, viscosity, and stability. During optimization, it was observed that several formulations had issues with stability, excipient incompatibility, and viscosity. Thus, the formulations with these issues were dropped and reformulated by changing the excipient concentration to achieve optimized formulations (Table A1, Table A2 and Table A3) with desired qualities for further studies.

**Table A1:** Optimization of cream formulations.

Components	Formulation Trial (%*w*/*w*)									
	1	2 (F1A)	3 (F1B)	4	5 (F2A)	6 (F2B)	7	8 (F3)	9	10 (F4)
Ibuprofen	3	3	3	3	3	3	5	5	5	5
Propylene glycol	15	15	15	-	-	-	-	-	-	-
Oleyl alcohol	-	-	-	5	5	5	-	-	-	-
Isopropyl Myristate	-	-	-	-	-	-	10	10	-	-
Transcutol	-	-	-	-	-	-	-	-	15	15
Mineral oil	10	10	10	-	-	-	7	7	-	-
White petrolatum	-	-	-	10	10	10	-	-	-	-
Polyethylene glycol 400	-	-	-	-	-	-	10	10	-	-
Medium chain triglycerides	-	-	-	-	-	-	-	-	10	10
Glycerol	-	-	-	-	-	-	-	-	3	5
Span 20	3.8	3.8	3.8	-	-	-	4.6	4.7	-	-
Kolliphor Polysorbate 80	3.3	3.3	3.3	-	-	-	2.1	1.3	-	-
Kolliphor CS 20	-	-	-	4.1	4.1	4.1	-	-	5	5
Glycerol monostearate	-	-	-	2.5	2.5	2.5	-	-	1.1	1.1
Cetyl alcohol	4	7	7	-	-	-	-	-	4	7
Stearyl alcohol	-	-	-	5	10	10	-	-	-	-
Cetostearyl alcohol	-	-	-	-	-	-	-	5	-	-
Sepineo SE68	3	5	5	-	-	-	-	-	-	-
Sepineo P600	-	-	-	-	-	-	4	4	-	-
Tefose 63	3	5	-	-	-	-	7	8	-	-
HPMC	-	-	-	-	1	-	-	-	-	-
Carbopol 974	-	-	-	-	-	-	-	-	0.5	0.5
Water	54.9	47.9	52.9	70.4	64.4	65.4	50.3	45	56.4	51.4
**Observation**	Emulsion was not stable	Thick white cream with no signs of separation	Less viscous white cream with no signs of separation	Less viscous cream with physical instability	Stable white cream with no signs of separation	Smooth white cream with no signs of separation	Physical instability was observed	Stable emulsion with no signs of instability	Physical instability was observed	Stable cream with no signs of separation

**Table A2:** Optimization of emulgel formulation.

Components	Formulation Trial (%*w*/*w*)		
	1	2	3 (F5)
Ibuprofen	3	3	3
Ethanol	10	20	30
Sepineo P600	4	4	4
Water	83	73	63
**Observation**	Physical instability. Precipitation of ibuprofen in the formulation	Slight precipitation of ibuprofen in the formulation	Translucent gel with acceptable viscosity

**Table A3:** Optimization of clear gel formulation.

Components	Formulation Trial (%*w*/*w*)									
	1	2	3	4	5	6	7	8	9	10 (F6)
Ibuprofen	3	3	3	3	3	3	3	3	3	3
Propylene glycol	15	5	15	15	15	15	15	15	15	15
Ethanol	-	-	-	-	10	10		10	10	10
Transcutol	5	15	15	15	15	15	15	15	15	15
HPMC	1.5	-	1.5	-	1.5	-	1.5	-	-	-
HPC	-	-	-	-	-	-	-	1.5	3	4
Carbopol 974	-	1.5	-	1.5	-	1.5	-	-	-	-
Polyethylene glycol 400	-	-	-	-	-	-	75.5	55.5	54	53
Water	75.5	75.5	65.5	65.5	55.5	55.5	-	-	-	-
**Observation**	Precipitation of drug was observed. Gel was not clear gel	Precipitation of drug was observed. Gel was not clear gel	Precipitation of drug was observed. Gel was not clear gel	Precipitation of drug was observed. Gel was not clear gel	Precipitation of drug was observed. Gel was not clear gel	Precipitation of drug was observed. Gel was not clear gel	HPMC was not dissolved in PEG 400	Gel was clear but not viscous	Gel was clear but not viscous	Clear gel with acceptable viscosity
